# Incidence of dementia and association with APOE genotype in older Cubans

**DOI:** 10.1590/S1980-57642014DN84000009

**Published:** 2014

**Authors:** Juan J. Llibre Rodríguez, Adolfo Valhuerdi Cepero, Isis Y. Sanchez Gil, Ana M. López Medina, Juan C Llibre-Guerra, Jorge J Llibre-Guerra, Beatriz Marcheco Teruel, Cleusa P Ferri, Martin Prince

**Affiliations:** 1 Medical University of Havana, Cuba; 2 Medical University of Matanzas, Cuba; 3 Institute of Neurology Havana, Cuba; 4 National Centre for Medical Genetics, Havana, Cuba; 5 Universidade Federal de São Paulo - Department of Psychobiology, São Paulo, Brazil; 6 Centre for Global Mental Health, Health Service and Population Research Department, Institute of Psychiatry, King's College London, UK

**Keywords:** dementia, epidemiological studies, incidence study, risk factors, ApoE, Latin America

## Abstract

**Objective:**

In an admixed population of older Cubans, the incidence and association of APOE and sociodemographic risk factors with dementia incidence was estimated.

**Methods:**

A single-phase survey (baseline) of all over 65-year-olds residing in seven catchment areas in Cuba (n=2944) was conducted between 2003 and 2007. Dementia diagnosis was established according to DSM-IV and 10/66 criteria. APOE genotype was determined in 2520 participants. An incidence wave was conducted 4.5 years after cohort inception in order to estimate incidence and associations with sociodemographic risk factors of the APOE ε4 genotype.

**Results:**

The incidence rate of DSM IV dementia was 9.0 per 1000 person-years (95% CI 7.2-11.3) and of 10/66 dementia was 20.5 per 1000 person-years (95% CI, 17.6-23.5). Older age, a family history of dementia and APOE ε4 genotype were independent risk factors for incident 10/66 dementia. APOE genotype was associated cross-sectionally with dementia prevalence, but the effect on the incidence of dementia was attenuated, and only apparent among those in the youngest age group.

**Conclusion:**

The incidence of dementia in the older Cuban population is relatively high and similar to levels reported in Europe and North-America. The study showed that the relationship between APOE ε4 and incident dementia is stronger in the younger-old than the older-old and that this change must be taken into account in models of dementia.

## Abstract (pt)

**Objetivo:**

Em uma população miscigenada de cubanos idosos, estimamos a incidência de demência e a associação entre o genótipo da APOE e os fatores de risco sociodemográficos na incidência de demência.

**Métodos:**

Realizamos uma pesquisa de uma fase (linha de base) de todos os idosos com mais de 65 anos residentes em sete áreas de Cuba (n=2944), de 2003 a 2007. O diagnóstico de demência foi estabelecido de acordo com os critérios do DSM-IV e do 10/66. O genótipo APOE foi determinado em 2520 participantes. Avaliação da incidência foi conduzida 4,5 anos após a linha de base, a fim de estimar a incidência e associações com fatores de risco sociodemográficos e o genótipo APOE ε4.

**Resultados:**

A taxa de incidência de demência foi de 9,0 por 1000 pessoas-ano (IC 95% 7,2-11,3) de acordo com o DSM-IV e de 20,5 por 1000 pessoas-ano (IC 95%, 17,6-23,5) de acordo com o 10/66. Idade avançada, história familiar de demência e genótipo APOE ε4 foram fatores de risco independentes para a incidência de demência de acordo com os critérios do 10/66. O genótipo APOE foi associado com a prevalência de demência em estudo transversal, mas o efeito sobre a incidência de demência foi atenuado, e apenas aparente entre aqueles na faixa etária mais jovem.

**Conclusão:**

A incidência de demência na população cubana mais velha é relativamente alta, semelhante às relatadas na Europa e América do Norte. O estudo mostra que a relação entre APOE ε4 incidente e demência é mais forte entre os idosos mais jovens e que esta alteração deve de ser considerada em modelos de demência.

## INTRODUCTION

By 2020, the Americas will have a population of 200 million older adults, with over half living in Latin American and the Caribbean. Population ageing is the major driver of the growing epidemic of chronic non-communicable diseases, concentrated in low- and middle-income countries (LMIC).^1,2^

Studies on the incidence of dementia are much less common than prevalence studies partially because of the considerable resources and time required for the former. Only a few incidence studies have been conducted in LMIC, which, generally, report lower incidence rates compared to high-income countries (HIC).^3-5^

The ε4 allele of the apolipoprotein-E gene has been the most consistently replicated genetic risk factor for dementia.^6,7^ In late onset sporadic as well as familial cases, which account for at least 95% of all cases, the apolipoprotein E (APOE) gene on chromosome 19 has been identified as a major risk factor.^7^ Most of the evidence suggests that this association is less consistent for individuals >80 years of age, may be stronger in women than in men, and also differs between ethnic groups.^4,8^ So far, however, African-Americans, other populations of west African ancestry, and Hispanics have shown relatively weak and inconsistent associations with AD, despite those with African ancestry tending to have a higher prevalence of the risk-conferring APOE ε4 allele.^7,8^

Cuba is a middle-income country with a highly admixed and rapidly ageing population of 11.3 million. By the year 2020 Cuba will be the country in Latin America with the highest proportion of older adults (25% aged 60 years and over).^9^

The main aims of this study were to describe dementia incidence and the association between APOE ε4 carriers and sociodemographic risk factors with dementia incidence among older Cubans.

## METHODS

Study design. The Cuban site of the 10/66 study involved a cohort of adults aged >65 years in selected areas of the provinces of La Habana and Matanzas. The 10/66 protocol has been published elsewhere.^10,11^

A cross-sectional study has also been published.^12^ Briefly, a single-phase survey (baseline) screening all over 65-year-olds residing in seven catchment areas in Cuba (n=2944) between 2003 and 2007 was performed. A total of 320 cases of dementia were diagnosed, representing a dementia prevalence of 6.4% according to the DSM-IV criteria and 10.8% according to the 10/66 criteria.

**Figure 1:**
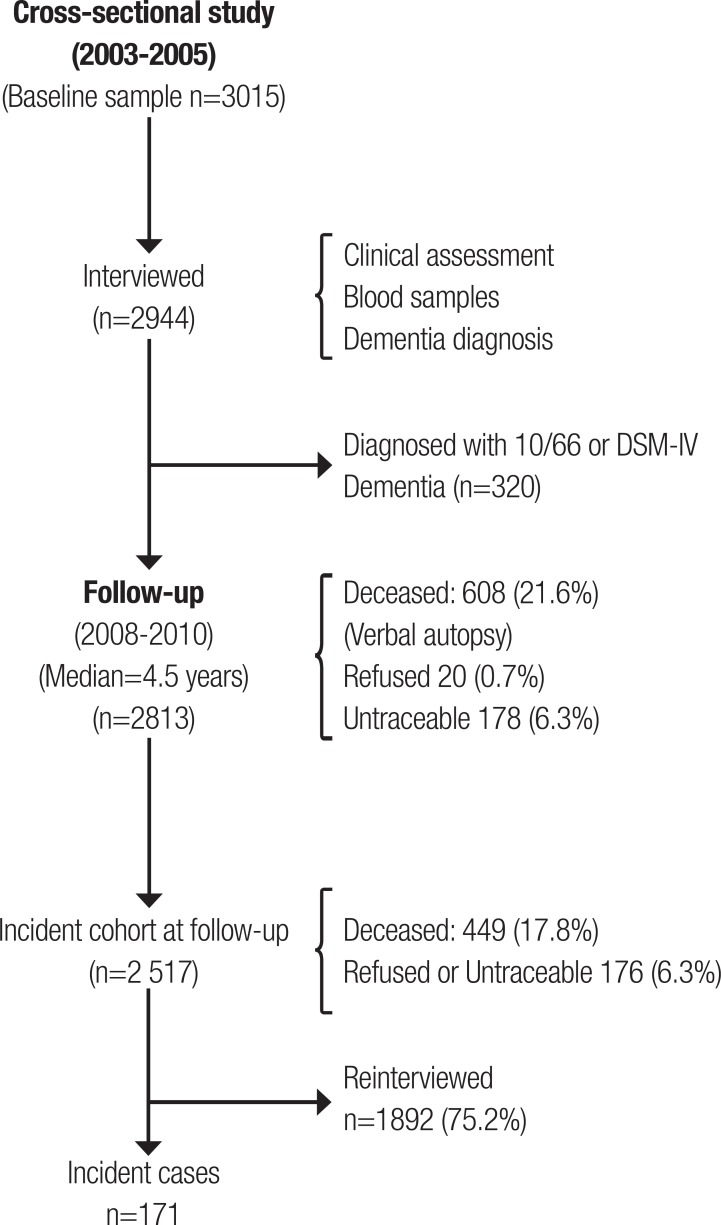
The Havana and Matanzas prevalence and incident study sample.

The incidence phase was conducted from 2008 to 2010 with a median follow up of 4.5 years after the baseline interviews. Of the 2,944 baseline sample participants, 131 from one polyclinic were not followed up because of logistic difficulties; therefore only 2,813 were eligible for the incidence phase. Of these, 2007 (71.3 %) were successfully re-interviewed. Over the period, there were 608 (20.6%) deaths and 198 (6.7%) subjects were lost to follow-up. The cohort for the analyses of dementia incidence was defined as all those who were free of dementia (either DSM-IV or 10/66 dementia) at baseline (n=2517) (see Figure 1).

The 10/66 protocol was applied;^10^ it included a structured participant interview covering sociodemographic characteristics, health status, behavioral and other risk factors; a physical and neurologic exam; and interview of a reliable informant. Interviews and instrument application were carried out by trained medical specialists at participants' homes, in sessions lasting 2-3 hours on average, which included interviewing of participants, physical examination and phlebotomy, plus an informant interview. Data were collected directly onto laptop computers using computerized Spanish questionnaires driven by Epidata software, including conditional skips and interactive checking.

For the purposes of this study, the following variables were considered:

### Outcome - The diagnosis of dementia

Dementia was diagnosed according to the 10/66 criteria and diagnostic algorithm, validated in 26 culturally heterogeneous countries, including Cuba.^11,13^ and according to DSM-IV criteria.^14^

### Main exposures

- Sociodemographic characteristics: age, sex, marital status, education, number of assets in the household, food insecurity were collected with a standardised questionnaire.
- Behavioral risk factors: Smoking status included smoker, ex-smoker and non-smoker. as well as lifetime smoking. Alcohol use questions covered maximum number of units per week before and after the age of 65 years. The threshold for hazardous drinking was set at 14 units per week for women and 21 units for men.
- Health status.
- Diabetes mellitus diagnosis was reached in two ways: self-report that diabetes had been diagnosed by a physician, and / or fasting blood glucose >7mmol / L, confirmed on two different days.^15^
- Hypertension diagnosis based on self-report and / or by direct measurement of blood pressure. Systolic blood pressure >140 mm Hg and / or diastolic pressure of >90 mm Hg were considered hypertension, according to guidelines of the Joint National Committee on Prevention, Detection, Evaluation and Treatment of High Blood Pressure.^16^
- Stroke: self-report that stroke had been diagnosed by a physician.

Laboratory exams Blood samples from 2520 participants were tested for hemoglobin, haematocrit, mean cell hemoglobin, fasting blood glucose, lipid profile, vitamin B12, folic acid and thyroid hormones at the National Center of Medical Genetics in Havana. In addition, cell DNA was extracted and ApoE4 genotype determined by PCR, following the standard protocol for determination of the apolipoprotein E genotype and identification of the three alleles APOE ε2, APOE ε3 and APOE ε4.^17^

The same protocols for interviews and assessments were employed in both the longitudinal phase of the study and at baseline. Quality control procedures included repetition of 5% of interviews by a specialist from the research team.

**Ethics**. Informed written consent was obtained from participants or, if necessary, their caregivers. All data were kept confidential. The study protocol was approved by the Research Ethics Committee of the Medical University of Havana.

**Analysis**. Person-years at risk for the onset of the relevant dementia outcome (DSM-IV dementia or 10/66 dementia) were calculated as the interval between baseline and follow-up assessment, or the mid-point of this interval for those that were found to have developed dementia. Age-specific incidence (with Poisson standard errors and 95% confidence intervals) was estimated using the Open Epi online calculator http://www.sph.emory.edu/~cdckms/exact-rate.html) by sex and age in 5-year bands by dividing the number of cases by the number of person-years contributed in each age band. The strength of the association of age, sex, educational level, family history of dementia and APOE genotype (presence vs absence of an APOE ε4 allele) with the prevalence of dementia was examined using Poisson regression. The incidence of dementia was determined using Cox regression models (generating hazard ratios, approximating to incidence rate ratios) in the dementia-free at risk cohort, censoring those who had died at baseline and employing Stata's stcrreg command to implement a competing-risks regression based on Fine and Gray's proportional subhazards model.

Given that lipid levels and other cardiovascular risk factors were determined during the cross-sectional study in late-life and close to the clinical onset of dementia and that temporality cannot be established, the analysis included only those exposures of possible aetiologic significance. All models were controlled for the effects of age, gender and education.

## RESULTS

**General characteristics of the sample**. Sociodemographic characteristics are summarized in Table 1. Mean age at baseline was 75.1 (SD 7.0) years; 25.4% of the sample was aged 80 years or older, 64.9% were female and 8.9% were living alone. Levels of education were relatively high, with only 2.5% illiteracy and 16.9% having attained tertiary education. There was a high prevalence of cardiovascular risk factors and of chronic non-communicable disease; more than 40% of participants were current smokers, 73.9% of participants had been told that they were hypertensive, 18.5% had received a diagnosis of diabetes, and 7.8% reported a stroke diagnosed by a clinician.

**Table 1:** Baseline characteristics of sample stratified by follow-up status.

		Baseline sample (n=2944)	Incidence phase			
			Re-interviewed (n=2007)	Died (n=608)	Lost to follow-up (n=198)	p Value
Female (MV=0)		1904 (64.9%)	1332 (66.4)	365 (60.0)	139 (70.2)	P=0.005
Age (MV=7)	65-69	760 (25.8%)	607 (30.3)	59 (9.7%)	49 (24.7)	P<0.0001
	70-74	789 (26.8%)	578 (28.9)	114 (18.8)	55 (27.8)	
	75-79	639 (21.7%)	435 (21.7)	137 (22.6)	46 (23.2)	
	80+	749 (25.4%)	381 (19.0)	297 (48.9)	48 (24.2)	
Lives alone (MV=8)		261 (8.9%)	174 (8.7)	53 (8.7)	23 (11.6)	P=0.597
Marital status (MV=8)	Married	1271 (43.3%)	903 (45.1)	216 (35.8)	80 (40.4)	
	Widowed	928 (31.6%)	586 (29.3)	239 (39.6)	71 (35.9)	
	Separated/ Divorced	462 (15.7%)	334 (16.7)	78 (12.9)	36 (18.2)	
	Never married	275 (9.4%)	180 (9.0)	71 (11.7)	11 (5.6)	
Education (MV=8)	None	75 (2.5%)	42 (2.1)	26 (4.3)	5 (2.5)	
	Minimal	655 (22.2%)	422 (21.1)	166 (27.5)	31 (15.7)	
	Completed primary	979 (33.3%)	651 (32.5)	222 (36.8)	64 (32.3)	
	Completed secondary	728 (24.4%)	540 (27.0)	109 (18.1)	56 (28.30	
	Tertiary	499 (16.9%)	348 (17.4)	81 (13.4)	42 (21.2)	
**Socioeconomic indicators**						
Number of Assets (MV=8)	0-3	78 (2.6)	47 (2.4)	20 (3.3)	10 (5.1)	P=0.730
	4-5	951 (32.6)	630 (31.5)	232 (38.20	45 (22.7)	
	6+	1891 (64.8)	1323 (66.2)	355 (58.5)	143 (72.2)	
Food insecurity (MV=8)		140 (4.8%)	90 (4.5)	39 (6.5)	8 (4.0)	P=0.084
**Life style**						
Current smoker (MV=9)		563 (42.5%)	369 (40.8)	136 (47.4)	37 (45.7)	P=0.218
Hazardous drinker (MV=17)		105 (3.6%)	66 (3.3)	31 (5.1)	6 (3.0)	P=0.484
**CV diseases and risk factors**						
Hypertension (MV=4)		2 944 (73.9%)	1488 (74.3)	448 (73.7)	154 (77.8)	P= 0.661
Stroke (MV=6)		230 (7.8%)	113 (5.6)	88 (14.6)	15 (7.6)	P=0.751
Diabetes (MV=16)		543 (18.5%)	354 (17.7)	129 (21.5)	36 (18.2)	P=0.586

MV: missing values

There were no substantial differences in the characteristics of those interviewed at baseline and the subset successfully followed-up. However, there were statistically significant differences in gender, age and education between those successfully interviewed at follow up, those who died and those who were untraceable. Those who died were older, more likely to be women and have lower education (Table 1).

**The incidence of dementia**. In total, 2,517 out of the 2,813 participants interviewed at baseline and included in the follow-up phase were free of dementia and hence eligible for inclusion in the 'at risk' cohort at baseline. Of this cohort, 1892 (75.2%) were successfully traced and re-interviewed at follow-up. These participants contributed 8,679 person years of follow-up, with an average follow-up period of 4.5 years. Mean age at follow-up was 78.1 years, two-thirds were female and educational levels were relatively high, but 7.7% of participants reported illiteracy.

There were 170 incident cases of 10/66 dementia and 77 cases meeting criteria for DSM-IV dementia. Only one incident case of DSM-IV dementia did not meet 10/66 dementia criteria. The crude annual incidence rate for 10/66 dementia was 20.5 / 1000 per 1000 person-years (95% CI 17.6-23.8) whereas for DSM-IV dementia was 9.0/1000 person-years (95% CI 7.2-11.3) (Table 2). Incidence tended to be higher in women (21.9 / 1,000 person-years, 95% CI 18.2-26.2) than men (17.8, 95% CI 13.9-23.5) for 10/66 dementia, but similar according to DSM-IV, where incidence was slightly lower in women (9.1, 95 % CI 6.9-12.0) than in men (9.6, 95 % CI 6.6-14.0). Incidence of both dementia outcomes increased exponentially with increasing age (Table 2).

**Table 2:** Annual incidence rates (per 1000 person-years) for DSM-IV and 10/66 dementia criteria by sex and age.

Age group	Gender	10/66 dementia			DSM-IV dementia	
		Cases / years	Incidence (95 % CI)*		Cases / years	Incidence (95 % CI)*
65-69 n=587	Female	9 / 1803	5.0 (2.6-9.6)		5 / 1803	2.7 (1.2-6.6)
	Male	7 / 936	7.5 (3.6-15.7)		4 / 936	4.3 (1.6-11.4)
	Total	16 / 27	5.8 (3.6-9.6)		9 / 275	3.3 (1.7-6.3)
70-74 n=545	Female	32 / 1599	20.0 (14.5-28.3)		14 / 1599	8.8 (5.1-14.8)
	Male	12 / 903	13.3 (3.7-9.3)		9 / 903	10.0 (5.2-19.2)
	Total	44 / 250	17.6 (13.1-23.6)		23 / 2558	9.0 (6.0-13.4)
75-79 n=405	Female	32 / 1142	28.0 (19.8-39.6)		13 / 1142	11.4 (6.6-19.6)
	Male	15 / 577	26.0 (15.6-43.1)		8 / 577	13.8 (6.9-27.7)
	Total	47 / 172	27.3 (20.5-36.4)		21 / 178	11.8 (7.7-18.1)
80+ n= 309	Female	46 / 926	49.6 (37.2-66.3)		18 / 995	18.1 (11.4-28.7)
	Male	15 / 379	39.6 (23.9-65.6 )		5 / 401	12.4 (5.2-29.9)
	Total	61 / 131	46.7 (36.4-60.1)		23 / 140	16.5 (10.9-24.8)
All ages n= 1,886	Female	120 / 5484	21.9 (18.2-26.2)		50 / 5484	9.1 (6.9-12.0)
	Male	50 / 2807	17.8 (13.5-23.5)		27 / 2807	9.6 (6.6-14.0)
	Total	170 / 8292	20.5 (17.6-23.8)		77 / 8517	9.0 (7.2-11.3)

Table 3 gives the prevalence ratio (PR), hazard ratio (HR) and competing risk (SHR) estimates for sociodemographic factors (age, sex and education), familial and genetic factors (family history of dementia and APOE genotype). All analyses were controlled for age, sex, and education.

**Table 3:** Prevalence ratio, Hazard ratio and SubHazard Ratio (competing risk) with 95% confidence intervals for associations between 10/66 dementia and sociodemographic, familial and genetic risk factors, adjusted for age, sex and education.

Exposures	Prevalence Ratio (95% CI) (n=2910)	Hazard Ratio (95% CI) (n= 1852)	Competing risk - SHR (95% CI) (n=2302)
Age (per 5-year band)	1.99 (1.76-2.26) MV=15	1.80 (1.56-2.09) MV=9	1.56 (1.35-1.79) MV=11
Sex (Male vs. Female)	0.89 (0.72-1.12) MV=15	0.88 (0.62-1.24) MV=9	0.78 (0.55-1.09) MV=11
Education (per level)	0.80 (0.72-0.89) MV=15	0.93 (0.81-1.08) MV=9	0.95 (0.83-1.09) MV=11
Family history of dementia	1.61 (1.28-2.04) MV=18	1.45 (1.00-2.11) MV=10	1.49 (1.04-2.14) MV=14
APOE genotype (any APOE ε4 allele vs. none)	2.53 (2.02-3.17) MV=423	1.48 (1.00-2.24) MV=236	1.57 (1.05-2.37) MV=308

There was a significant association of increasing age (PR=1.99; 95% CI 1.76-2.26), family history of dementia (PR=1.61; 95% CI 1.28-2.04) and APOE ε4 genotype (PR 2.53; 95% CI, 2.02-3.17), with an increased prevalence of 10/66 dementia. Education level (PR 0.80; 95% CI 0.72-0.89) was inversely associated. Patterns of association with incident 10/66 dementia were somewhat different. The effect of increasing age seemed attenuated, particularly when the competing risk of death was accounted for in the analysis. The effect of one or two APOE ε4 alleles was also attenuated, and only statistically significant when the competing risk of dementia-free death was accounted for (SHR 1.57, 95% CI 1.05-2.37). Also, the inverse association with education was not apparent with respect to incident 10/66 dementia.

**Figure 2:**
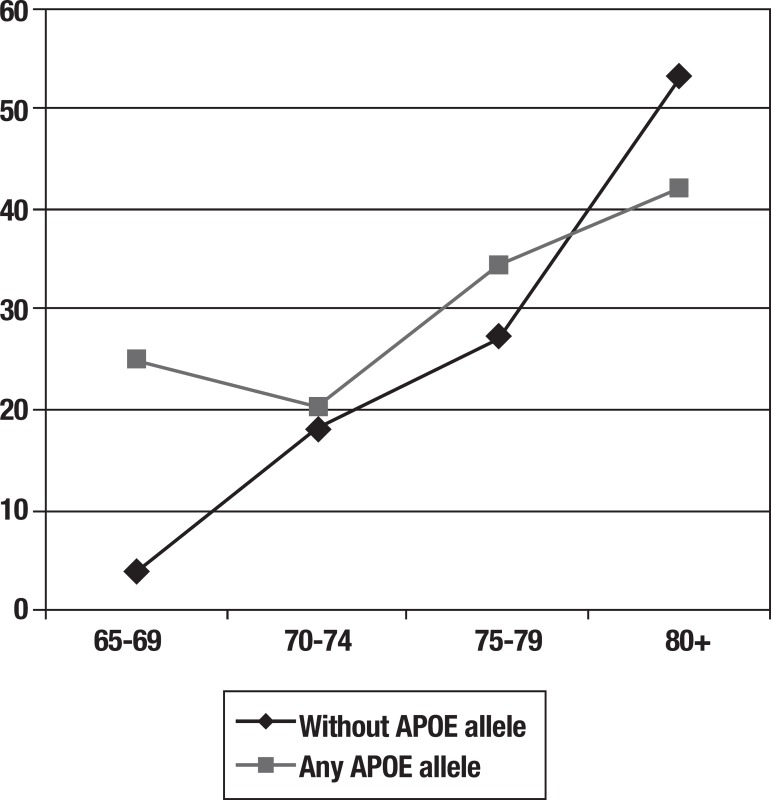
Age and incidence of dementia for participants with Apolipoprotein E4 compared to individuals without an Apolipoprotein E4 allele.

Table 4 compares the incidence rates of 10/66 dementia according to age group and APOE status. Incidence increases sharply with age for those with no APOE ε4 allele, but much less steeply for those with one or two APOE ε4 alleles. The effect of APOE genotype on dementia incidence appeared to be principally confined to the youngest age group. For participants aged 65-69 years with one or two APOE ε4 alleles, incidence rates for dementia were seven times higher than for participants without APOE alleles. Among those aged 80 years and over, dementia incidence was actually lower among APOE ε4 carriers than among non-carriers. The pattern is illustrated graphically in Figure 2.

**Table 4:** Incidence rates of dementia (per 1,000 person-years) by age group and APOE status.

Age group	Dementia Incidence rates (95%CI)	
	Any APOE4 allele	Without APOE4 allele
65-69	25.1 (13.1-48.5)	3.5 (1.6-7.3)
70-74	20.3 ( 9.7-42.6)	18.3 (13.1-25.5)
75-79	34.6 (16.5-72.6)	27.0 (19.5-37.5)
80 or older	42.1 (18.9-93.8)	53.3 (40.6-70.0)
Whole sample	27.7 (19.2-39.8)	21.1 (17.7-25.0)

The interaction of age with APOE genotype in the association with incident 10/66 dementia was confirmed in a model testing for the main effect of APOE genotype (any ε4 allele vs none), the main effect of age (linear effect per five year increment), and the interaction between the two, again controlling for sex and educational level. The interaction term was statistically significant (SHR 0.71, 95% CI 0.53-0.96), indicating a substantial progressive reduction in the effect of APOE ε4 with increasing age, from that estimated for the baseline age group (SHR 3.99, 95% CI 1.71-9.31). Likewise, the estimated effect of age for those lacking an APOE ε4 allele (SHR 1.47, 95% CI 1.31-1.64) was reduced in the presence of an ε4 allele to a SHR of 1.04.

The clear implication of this pattern of incidence with age, is that the age of onset of incident dementia cases is younger among those with one or more APOE ε4 alleles, compared with those lacking an ε4 allele.

## DISCUSSION

This study corroborates that dementia is an important growing health problem for Cuba. The major strength of our study is the standardised design and assessment procedures, in a large representative catchment area sample, with a high response rate: 97.6 % in the cross sectional study and 75.8% in the incidence phase.

The diagnosis of dementia was reached according to a protocol developed by the 10/66 group using a computerized algorithm. In a recent publication^18^ we have shown that 10/66 dementia corresponded more closely to Cuban clinical dementia diagnoses than did the more restrictive DSM-IV criterion.

The age-specific incidence of 10/66 dementia in Cuba was consistently higher than that of DSM-IV dementia. In a previous study, we have noted that DSM-IV dementia criterion underestimates the true prevalence of dementia in developing countries due to difficulties defining and ascertaining decline in intellectual function and occupational impairment.^12^

Few incidence studies have been conducted in low- and middle-income countries, and the current study is one of the largest conducted to date in a low- or middle-income country; in Ballabgarh, India, nine incident cases were identified with 1,160 person-years of follow-up;^19^ in Catanduva, Brazil, 50 incident cases were detected with 3,623 person-years of follow-up.^3^ Other studies were performed in Ibadan, Nigeria (2,459 at risk and 70 incident cases)^4^ and Beijing, China (825 at risk and 13 incident cases),^5^ although person-years of follow-up were not clearly specified.

The crude annual incidence rate for 10/66 dementia detected in the present study was very similar to that found in the Canadian Health and Aging Study (20), and slightly higher than that reported in the MRC Cognitive Function and Ageing Study (MRC CFAS) in England.^21^ Nevertheless, according to DSM-IV criteria our estimates were 9.0 / 1000 person-years, roughly half the rates observed in the Canadian and English studies, both of which used DSM-IV criteria. However, to estimate the incidence of DSM-IV dementia, we excluded all subjects with 'any dementia', i.e. either DSM-IV or 10/66 dementia, from the baseline 'at risk' cohort. This decision is justifiable on the grounds that there is considerable accumulated evidence supporting the validity of the 10/66 dementia diagnostic criterion.^15^ However, for the purposes of comparison with other studies, it might be appropriate to consider meeting criteria for 10/66 dementia, but not for DSM-IV dementia, as still 'at risk' for the latter outcome. In the Cuban sample, the annual incidence rate for DSM-IV dementia among those in this group was 154.6 per 1000 person-years (95% CI 103.9-221.8). After including this group in the 'at risk' cohort, the overall incidence rate for DSM-IV dementia increased from 9.0 to 12.0 per 1000 person-years (95% CI, 9.8-14.4)

We found a strong association between APOE genotype and the prevalence of both 10/66 and DSM-IV dementia, with effect sizes very similar to those reported in other settings.^22-25^

However, the association between APOE genotype and incident dementia was, in comparison, greatly attenuated. The reason for this much reduced strength of association with incident as opposed to prevalent dementia is not immediately clear, and may be complex. One possible explanation, that APOE ε4 prolongs survival with dementia rather than increasing its incidence, seems unlikely given the weak effect of APOE genotype on overall survival, and the absence of an interaction between dementia status and APOE genotype as risk factors for mortality. A likelier explanation is suggested by the strong interaction observed between age and APOE genotype in risk for onset of 10/66 dementia, where the increased risk conferred by the APOE ε4 allele appeared to be confined to individuals in the younger-old age groups . Further analysis revealed a very strong effect of APOE genotype on age of onset, with APOE ε4 allele carriers having a mean age of onset 4.6 years earlier than those lacking an APOE ε4 allele but who went on to develop dementia. Both the concentration of risk among the younger-old, and the younger age of onset among APOE ε4 carriers were noted 15 years ago in clinical samples by members of the NIMH genetics initiative.^26^ A similar phenomenon was illustrated in the US Cache County study, where APOE genotype was found to influence age of onset, but not lifetime (up to 100 years) cumulative risk of dementia, which proved similar (72%) for those with and without APOE ε4 alleles.^27^ It may be the case that our prevalence study had already captured much of the (earlier) cumulative incidence in those who had elevated risk for early incidence by carrying one or more APOE ε4 allele. Set against this, while there have been very few previous population-based studies of the effect of APOE genotype on the incidence of dementia, findings from the UK MRC-CFAS study do indicate a robust and sizeable increased relative risk.^28^

In conclusion, the incidence of dementia in the older Cuban population is relatively high and similar to incidences reported in Europe and North-America. Older age, a family history of dementia and APOE ε4 genotype were independent risk factors for incident 10/66 dementia. The study showed that the relationship between APOE ε4 and incident dementia is stronger in the younger-old than the older-old and that this change must be taken into account in models of dementia .
